# A Rare Case of Diffuse Alveolar Hemorrhage Secondary to Acute Pulmonary Histoplasmosis

**DOI:** 10.1155/2015/821749

**Published:** 2015-09-08

**Authors:** Kunal Grover, Hadeel Zainah, Shubhita Bhatnagar, Tricia Stein

**Affiliations:** ^1^Internal Medicine Department, Henry Ford Health System, Detroit, MI 48202, USA; ^2^Infectious Diseases Division, Henry Ford Health System, Detroit, MI 48202, USA

## Abstract

Diffuse alveolar hemorrhage (DAH) is a rare presentation of acute pulmonary histoplasmosis. While histoplasmosis has been reported to cause hemoptysis and alveolar hemorrhage in children, the English language literature lacks any adult case reports documenting this association. We report a case of pulmonary histoplasmosis where the initial presentation was pneumonia with a subsequent diagnosis of DAH.

## 1. Introduction

Histoplasmosis is a disease caused by the fungus* Histoplasma capsulatum* [1] which can present with a varying severity of illness ranging from nonspecific flu-like symptoms to disseminated disease with multiorgan failure. Pulmonary presentations commonly include acute pneumonia with lung consolidation, chronic cavitary pulmonary histoplasmosis, and pleural effusion. Complications from pulmonary histoplasmosis can include pericarditis, arthritis, erythema nodosum, mediastinal fibrosis, mediastinal lymphadenitis, mediastinal granuloma, and pulmonary nodules. Other nonpulmonary manifestations can include central nervous system histoplasmosis and progressive disseminated histoplasmosis.

DAH is a rare entity that is associated with histoplasmosis. Current literature lacks clinical case reports illustrating this association in adults. As a result of this rare presentation, a high clinical suspicion should be maintained to include* H. capsulatum* in the differential diagnosis of DAH in the appropriate clinical settings.

## 2. Case Description

**Figure 1:**
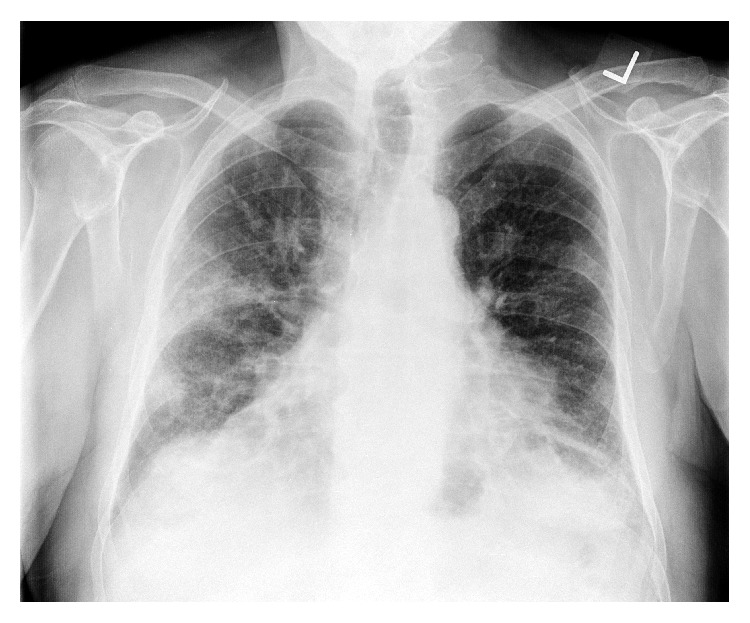
Chest X-ray 5-6 weeks before admission showing bilateral patchy lung opacities.

The patient is a 71-year-old Caucasian male who presented with a chief complaint of shortness of breath (SOB), productive cough, and dyspnea on exertion without fever. His past medical history was remarkable only for a remote history of smoking, tonsillectomy, and urinary retention. His family history was positive for Wegener's granulomatosis in a sibling and lung cancer in a parent. The patient lived in Michigan and frequently traveled to Chicago/Illinois area. Prior to admission, the patient had been seen by his primary care physician over the preceding 5-6 weeks for similar complaints. The patient's initial chest radiograph in the outpatient setting showed patchy bilateral airspace opacities (Figure 1). He was treated for a suspected community-acquired pneumonia (CAP) with a course of azithromycin and a subsequent course of moxifloxacin, with no clinical or radiologic improvement.

**Figure 2:**
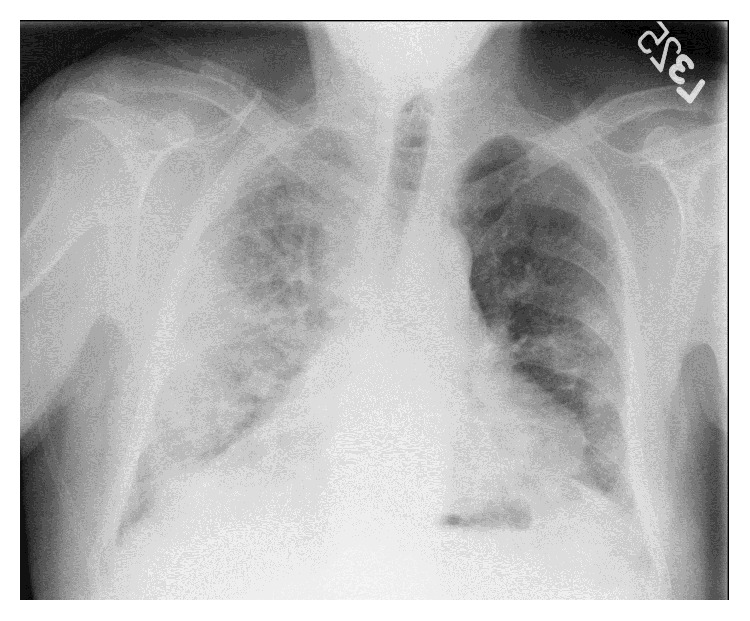
Chest X-ray on admission showing worsening bilateral lung opacities.

**Figure 3:**
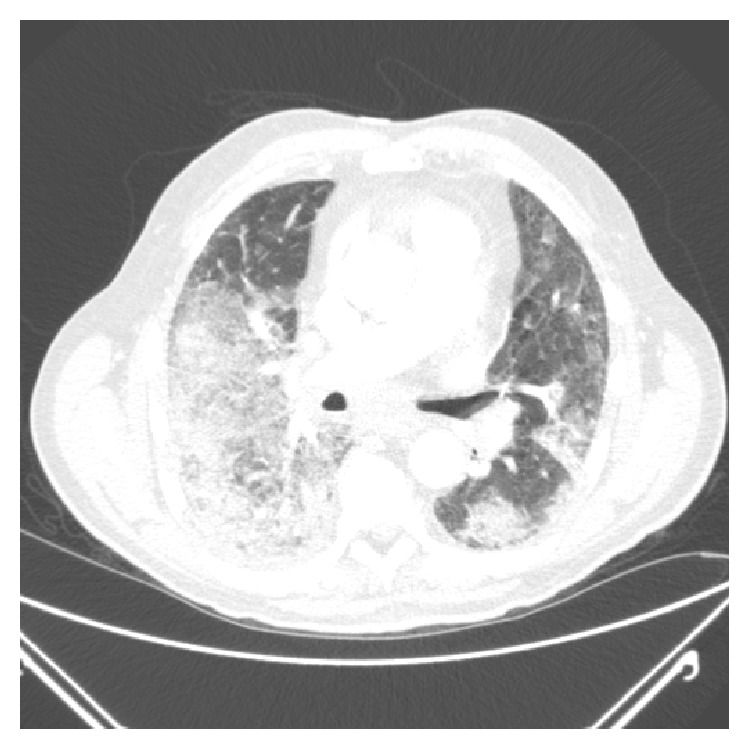
Computed tomography on admission showing bilateral pulmonary opacities.

Upon his arrival to hospital, the patient was found to have moderate respiratory distress. His vital signs showed a temperature of 36.1°C, a blood pressure of 171/88 mmHg, a heart rate of 102/min, a respiratory rate of 24/min, and an oxygen saturation of 86% on room air. On physical exam, the patient had bilateral wheezes and rhonchi and he was using accessory muscles to breathe. His cardiac exam revealed regular rhythm and tachycardia with no murmur. The patient was started on ceftriaxone and azithromycin in the Emergency Department. His initial laboratory workup showed a white blood cell count of 15.8 K/*μ*L (3.8–10.6), hemoglobin of 15.0 g/dL (13.5–17), platelet count of 220 K/*μ*L (150–450), and a creatinine of 1.0 mg/dL (0.8–1.3). A chest radiograph was remarkable for worsening airspace disease in the right lower lung and the central left lung (Figure 2). A computed tomography scan of the chest demonstrated diffuse lung disease, a small right pleural effusion, and enlarged intrathoracic and lower cervical lymph nodes (Figure 3). The patient's respiratory status continued to decline and he eventually required mechanical ventilation. The antimicrobial coverage was broadened to include vancomycin to cover potential methicillin-resistant* Staphylococcus aureus* and cefepime for Gram-negative coverage. A tapering dose of methylprednisolone was started for a concern of possible acute interstitial pneumonia or Wegener's disease given the patient's family history.

An extensive clinical workup was initiated. A bronchoscopy and bronchial alveolar lavage (BAL) were done and demonstrated DAH. The sample obtained was also noted to have vegetable material and grew alpha-hemolytic* Streptococcus *and* Candida albicans*; cytology was negative for malignant cells. Viral respiratory cultures for* influenza*,* parainfluenza*, respiratory syncytial virus, and* adenovirus* were all negative in the BAL, as was the* Pneumocystis jirovecii* antigen. Multiple bacterial and fungal blood cultures were negative. A nasopharyngeal swab was negative for* influenza* and* Bordetella pertussis *serology and urinary* Legionella pneumophila* antigen were negative as well. Furthermore, antineutrophil cytoplasmic antibody (C-ANCA and P-ANCA) and antinuclear antibody (ANA) were all negative.

The patient began spiking high-grade fevers (39.5°C) and metronidazole was added for concern of aspiration. The patient also developed bilateral lower extremity deep vein thromboses. A filter was placed in the inferior vena cava.

The patient continued to be febrile and developed thrombocytopenia (platelet count: 53 K/*μ*L) and his renal function declined (creatinine: 3.9 mg/dL). The* Histoplasma* urinary antigen was positive (4.3 EIA Units, normal value <2.0 EIA Units) and the (1-3)-B-D-glucan assay was positive (280 pg/mL, normal value <60 pg/mL). Subsequently, IV liposomal amphotericin B was initiated (5 mg/kg/day). The patient's methylprednisolone was maintained. The diagnosis of acute pulmonary histoplasmosis was made based on the geographic location, the radiological findings, the clinical findings, and the positive* Histoplasm*a urinary antigen. Serum* Histoplasma* antibodies obtained were negative at that time.

The hospital course was complicated by irreversible ischemia of the right lower extremity due to a thromboembolic event in the right peroneal, anterior tibial, and posterior tibial arteries. He eventually underwent an emergent thromboembolectomy and fasciotomy and was given intra-arterial tissue plasminogen activator (t-PA). A transthoracic echocardiogram did not reveal an evidence of valvular vegetations. During his stay, the patient required hemodialysis, higher ventilatory support, and blood transfusion (hemoglobin: 5.0 g/dL). The patient's right foot became dusky and the abdomen became distended. The patient's clinical condition continued to deteriorate, and he expired.

## 3. Discussion

Histoplasmosis is a disease caused by the fungus* H. capsulatum* with varying clinical presentations and severity of the illness [1].* H. capsulatum* is found in soil and material contaminated with bird or bat droppings often associated with caves, bats, and bird roosts [1].

Histoplasmosis infection tends to occur within two weeks after an inhalation exposure to* H. capsulatum*. The acute phase of infection is characterized by nonspecific flu-like symptoms and cough, often with a normal appearing CXR [1]. The severity of the disease varies depending on the exposure intensity, the pathogen inoculum, host immunity, and the presence of underlying structural lung disease [2].

Histoplasmosis by definition includes primary pulmonary histoplasmosis, with acute or chronic cavitary forms, progressive disseminated histoplasmosis with multiorgan involvement, and central nervous system (CNS) histoplasmosis. Other manifestations associated with histoplasmosis have been reported as previously mentioned [3].

DAH is a severe and life-threatening acute condition associated with bleeding into the alveoli related to the breakdown in the alveolar-capillary basement membrane secondary to an injury or inflammation of the blood vessels. The differential diagnosis for DAH is broad and can broadly be categorized in the following groups: vasculitis or capillaritis, “bland” pulmonary hemorrhage, and alveolar bleeding associated with another process or condition [4]. Capillaritis is a pathological process characterized by neutrophilic infiltration of the pulmonary interstitium. Bland pulmonary hemorrhage is characterized by bleeding into the alveolar spaces without associated vasculitis or capillaritis [4]. Our patient was diagnosed with DAH based of diffuse infiltrates on imaging, hemoglobin drop (15 to 5 g/dL), and bronchoscopy findings of blood in the alveoli. Patients with DAH generally present with nonspecific signs and symptoms. In patients with DAH, a high clinical suspicion should be maintained to include the possible diagnosis of histoplasmosis in the appropriate clinical setting.

Histoplasmosis has been previously reported as an etiology for hemoptysis and pulmonary hemorrhage [5] but, to our knowledge, no cases have been reported in the adult literature.

The diagnosis of Histoplasmosis requires a good clinical history, physical exam, imaging studies, and laboratory studies including microbiological, serological, and histopathological testing [6].

*Histoplasma* antigen can be detected in the urine and/or serum. Antigenuria was detected in up to 91.8% of the disseminated cases with higher detection rates when using the newer generation testing methods, in immunocompromised patients, and in patients with higher antigen titers [6]. Antigenuria is also detected at higher rates in patients with severe disease as compared to mild disease (100% versus 78.7%, resp.), as well as in patients with disseminated histoplasmosis infection [6]. Antigen detection is helpful in making the diagnosis before the antibodies turn positive in the serum [7], as seroconversion might take up to 3 months after acute pulmonary histoplasmosis [7].

In our case, imaging studies in combination with bronchoscopy led to the diagnosis of DAH. Extensive workup failed to identify a viral cause or bacterial cause other than alpha-hemolytic* Streptococcus* and rheumatologic investigation did not support the diagnosis of Wegener's disease. The patient's urinary* Histoplasma* antigen test was positive, thus establishing the diagnosis of acute pulmonary histoplasmosis in the absence of positive serum antibodies; this dissociation is explained by the earlier positivity of the antigen test before seroconversion.

Most* H. capsulatum* infections are self-limited and resolve without antifungal therapy. Current clinical practice guidelines from the Infectious Diseases Society of America (IDSA) recommend various treatment regimens depending on the type of disease [1]. Definitive indications for antifungal therapy include acute diffuse pulmonary infection with moderate-to-severe symptoms, chronic cavitary pulmonary infection, progressive disseminated infection, and CNS infection [1]. Other potential indications for antifungal therapy include acute focal pulmonary infection, asymptomatic cases, mild symptoms that persist for >1 month, mediastinal lymphadenitis, mediastinal granuloma, or inflammatory syndromes treated with corticosteroids [1]. Clinical conditions typically not recommended to be treated with antifungal therapy include mediastinal fibrosis, pulmonary nodules, broncholithiasis, and presumed ocular histoplasmosis syndrome [1].

The standard treatment for moderately severe-to-severe acute pulmonary disease is lipid amphotericin B (AmB) (3.0–5.0 mg/kg daily) or deoxycholate AmB (0.7–1.0 mg/kg daily) for 1-2 weeks, followed by itraconazole (200 mg twice daily for a total of 12 weeks) and methylprednisolone (0.5–1.0 mg/kg daily intravenously for 1-2 weeks) [1]. Similarly, the treatment of progressive disseminated histoplasmosis that is moderately severe-to-severe is liposomal AmB (3.0 mg/kg daily), AmB lipid complex (5.0 mg/kg daily), or deoxycholate AmB (0.7–1.0 mg/kg daily) for 1-2 weeks, followed by itraconazole (200 mg twice daily for at least 12 months) [1].

Amphotericin B in any formulation is rarely used as a single agent therapy and most patients are switched to itraconazole for the remainder of the therapy. In patients in whom the use of itraconazole is contraindicated or not tolerated, other azoles may be used as second-line therapies. Fluconazole has been used successfully as an alternative therapy to itraconazole. Furthermore, there have been reported cases of the newer azoles, posaconazole, and voriconazole, successfully treating various types of histoplasmosis infections [8]. Antigenuria can be used to follow the response to treatment; if antigen levels do not decline, it may indicate a treatment failure. Levels of urine antigen should be checked before treatment is begun, at 2 weeks and 1 month of treatment, every 3 months thereafter during the course of therapy, and at 6 months after the treatment is completed [1]. Decreasing antigen levels indicate response to treatment, while increasing levels often indicate possible relapse. The persistence of low-level antigenuria does not require extending the treatment course if the patient has clinically responded to therapy and received adequate duration of therapy.

Our patient was started on amphotericin B therapy (liposomal AmB: 5 mg/kg/day) with an initial improvement and resolution of his fever. Lipid formulation of amphotericin B was selected given the recent renal impairment to minimize additional nephrotoxicity. In addition, he was continued on treatment with methylprednisolone. Other events complicated the hospital course and the patient expired.

This clinical case report illustrates the rare association between* H. capsulatum* and DAH and the first adult case reported in the English language literature to our knowledge. In light of the potential life-threatening nature of this association,* H. capsulatum* should be considered in the appropriate epidemiological and clinical settings.
